# Fracking and infant mortality: fresh evidence from Oklahoma

**DOI:** 10.1007/s11356-019-06478-z

**Published:** 2019-10-11

**Authors:** Nicholas Apergis, Tasawar Hayat, Tareq Saeed

**Affiliations:** 1 University of Derby, Derby, UK; 2 King Abdulaziz University, Jeddah, Saudi Arabia; 3 Quaid-I-Azam University, Islamabad, Pakistan

**Keywords:** Fracking, Infants’ health at birth, Drinking water, Oklahoma, I10, I18, Q52, Q53

## Abstract

This paper explores the impact of shale gas and oil fracking wells on infants’ health at birth across Oklahoma counties. The empirical analysis makes use of the Dumitrescu-Hurlin causality test, as well as the (long-run) Pooled Mean Group method. The results clearly document that there is a unidirectional relationship between fracking activities and three alternative indexes of infants’ health at birth, as well as a significant impact of fracking on infants’ health indicators. In addition, the results illustrate the substantial role of fracking through the drinking water quality channel.

## Introduction

Hydraulic fracturing (fracking) has been an important innovation in the energy sector, especially after 2006 in the case of the Oklahoma state. U.S. production of oil and natural gas has increased dramatically, leading to abruptly lower electricity prices, stronger energy security, lower carbon emissions, and better air pollution. As a result, thousands of fracking wells have been drilled from Pennsylvania to Colorado to Texas and to Oklahoma. Nevertheless, this innovative drilling activity has raised serious concerns about a number of negative impacts. In particular, many communities have reached very different conclusions about the benefits and costs. On the benefit side, there have been recent studies which find that fracking increases economic activity, employment, income, and housing prices (Bartik et al. 2018; Apergis 2019). However, these activities are characterized by the presence of certain costs; at the same time, a rising concern is whether fracking wells can contaminate water resources while negatively impacting the public health of exposed populations (Finkel and Hays 2016); other studies have also highlighted elevated levels of contaminants in drinking wells located near fracking wells (Hildenbrand et al. 2015).

Health impacts have been ignored in previous studies; therefore, this work will attempt to explore whether taking explicitly into consideration the health factor could likely alter the net benefits associated with fracking. In particular, the empirical analysis will investigate how fracking across Oklahoma counties affects infants’ health conditions born to mothers living up to certain distances from a fracking well. Such fracking activities could affect public health through the channels of water quality and air pollution. Our study focuses on infants’ health at birth across Oklahoma’s counties while also considering the mechanism of drinking water as a potential driver of such association. Currie et al. (2017) explored a sample of 1.1 million births in Pennsylvania, spanning the period 2004 to 2013, to investigate how infants born to mothers living at different distances from active fracking sites are affected from such fracking activities; the empirical findings illustrate that such activities have a substantial effect on the fetus, which is vulnerable to certain pollution exposures, leading to low birth weight. Similar results were also received by Hill (2013). McKenzie et al. (2014) also find an escalated risk of birth defects for babies born near fracking wells. Their study investigates potential links between maternal residential proximity to active fracking wells and birth outcomes by considering 124,842 births between 1996 and 2009 in rural Colorado. The scarcity of more studies on the nexus between infants’ health problems and fracking activities, as well as the absence of any relevant study for the state of Oklahoma (a significant producer of fracking oil and natural gas), signifies the novelty of this work.

The novelties of this work are as follows: first, this work was never performed for the case of Oklahoma counties. Second, the analysis examines not only the impact on infants’ low birth weight status but also on their general health picture. Third, the analysis is implemented for various distances of maternal residents from the fracking wells. Finally, it explicitly considers the mechanism of drinking water as a potential driver of the association between fracking and infants’ health at birth.

## Data

Birth data are available on mothers’ residential locations. The empirical analysis is based on 590,780 births across all 76 Oklahoma counties, spanning the period 2006–2017, given that the fracking boom occurred in the year 2006, which characterizes the starting point of our sample. Birth certificates were obtained from the Oklahoma Health Department. They inform on birth weight, weight below normal figures (i.e., < 2500 g), the mother’s race (*D* = 1 for White; *D* = 2 for Afro-American; *D* = 3 for Asian; *D* = 4 for Hispanic; and 0 otherwise), mother’s education (*D* = 1 < high school; *D* = 2 high school; *D* = 3 college; *D* = 4 higher than college; and 0 otherwise), mother’s age (*D* = 1, < 20; *D* = 2, 20–30; *D* = 3, 30–40; and 0 otherwise), child parity (*D* = 1 first; *D* = 2 second; *D* = 3 third; *D* = 4 higher; and 0 otherwise), and mother’s residence location which is useful for estimating the distance from the fracking well. After filtering with missing information, we end up with 556,794 birth observations. In addition, the active drilled oil and natural gas wells in which fracking is applied measures shale gas and oil (fracking) activities, which provide a complete (active) set of shale gas and oil (fracking) drilling process. The data were collected from the Oil and Gas Division of The Oklahoma Corporation Commission. For each well, we got information on its location in the country. The dependent variable was tested through three alternative definitions: infant’s total weight, low weight (< 2500 g), and a composite health index that explicitly informs on multiple idiosyncratic characteristics of the infant (Kling et al. 2007; Anderson 2008). It has been generated as a combination of birth weight and other indicators in relevance to low birth weight, prematurity (gestation < 37 weeks), any congenital anomalies, plus any other abnormal condition. The higher the index, the more positive picture of the infant’s health at birth is. These data are measured as the mean over the standardized outcomes and were obtained from Oklahoma State Department of Health. The literature has also used the infants’ mortality rate as an independent variable, but this type of data could not be obtained by the authors.

To match births to fracking wells, the analysis uses the zipcode of the mother’s residence along with the distance from the fracking well. The distances were determined through the Resource for the Future’s Center for Energy Economics and Policy that uses geographic information system (GIS) technology. In that sense, the analysis divided the drilling wells that are within 1 km, 5 km, 10 km, and 20 km from mothers’ residence. As a result, we got 121,862 births within 1 km; 148,783 within 5 km; 157,664 within 10 km; and 128,485 within 20 km from the wells.

## Modeling methodology

The model specification yields:

$$ {b}_{it}={\alpha}_i+{c}_1{X}_{it}+{c}_2{\mathrm{Distance}}_{it}+{e}_{it} $$

where *b*_it_ is the maternal birth *i* at time *t*, *α*_*i*_ indicates county fixed effects, *X*_*it*_ shows a vector of maternal idiosyncratic characteristics, and *e*_*it*_ is the error term. The control variable vector contains information on the mother’s race, mother’s education background, mother’s age, and children’s parity. Distance is defined as the number of (fracking) wells within 1 km from the mother’s residence, or 1–5 km, or 5–10 km, and 10–20 km. One of the referees has recommended that additional variables that could be considered are the mother’s drinking and smoking habits, substance abuse, obesity, and a variable that dictates for how long has the mother lived in proximity to the drilling site. In addition, the literature has put forward other (mostly social determinants) factors that have an impact on infants’ birth rates, such as the material living and working conditions, as well as the social conditions in which people are born, live, and work, certain structural drivers, i.e., residential segregation, social capital/cohesion, and behavioral factors, such as biological and psychosocial factors, e.g., social support (Boerma and Bicego 1993; Sullivan et al. 1994; Crémieux et al. 1999; Gokhale et al. 2002; Hanmer et al. 2003; Nixon and Ulmann 2006; Pradhan and Arokiasamy 2010; Mathew 2012; Chalasani and Rutstein 2014; among others). However, the authors have been unable to track and obtain this type of variables from the data sources the paper has employed.

Estimates are drawn on Pooled Mean Group (PMG) estimators, developed by Pesaran et al. (1999) and with reference to long-run estimates. These PMG estimates presume that the long-run coefficients are equal across groups, while the constants can be different. In addition, the direction of causality between fracking wells and infants’ health at birth is investigated with the assistance of the Dumitrescu-Hurlin test (Dumitrescu and Hurlin 2012). This test investigates the homogenous non-causality hypothesis by considering both the heterogeneity of the estimates and their causal association. Testing the null hypothesis, the authors use the average of all individual Wald statistics.

## Empirical analysis

Before presenting the primary results, the analysis reports a second-generation panel unit root test so as to determine the degree of integration of the respective variables. The Pesaran (2007) panel unit root test does not require the estimation of factor loading to eliminate cross-sectional dependence. The null hypothesis supports the presence of a unit root. The results are shown in Table 5 in the Appendix and reject the presence of a unit root across all panel variables that contain a continuous presence. Table 1 reports the PMG results. There are three model specifications associated with alternative definitions of infants’ health at birth; column (1) reports the results in relevance to the distance of the well < 1 km, column (2) to the distance between 1 and 5 km, column (3) to the distance between 5 and 10 km, and column (4) to the distance between 10 and 20 km.

**Table 1:** Estimates of fracturing on infants’ health (2006–2017)

Variables	0–1 km	1–5 km	5–10 km	10–20 km
Dependent variable: total birth weight				
Distance	− 0.084***	− 0.078*	− 0.048	− 0.025
	[0.00]	[0.07]	[0.14]	[0.29]
Mother’s age	− 0.279**	− 0.256**	− 0.298**	− 0.247**
	[0.03]	[0.03]	[0.02]	[0.03]
Mother’s education	0.349***	0.388***	0.402***	0.375***
	[0.00]	[0.00]	[0.00]	[0.00]
Mother’s ethnicity/race	0.062	0.059	0.060	0.064
	[0.25]	[0.27]	[0.26]	[0.24]
Child parity	0.344***	0.677***	0.058**	0.651*
	[0.00]	[0.01]	[0.04]	[0.07]
*R*-squared adjusted	0.62	0.57	0.38	0.34
Dependent variable: low birth weight				
Distance	0.056***	0.046***	0.039*	0.014
	[0.00]	[0.01]	[0.10]	[0.18]
Mother’s age	0.224**	0.218**	0.229**	0.234**
	[0.03]	[0.04]	[0.03]	[0.02]
Mother’s education	− 0.914***	− 0.866***	− 0.914***	− 0.862***
	[0.00]	[0.00]	[0.00]	[0.00]
Mother’s ethnicity/race	0.047	0.051	0.048	0.046
	[0.29]	[0.28]	[0.29]	[0.30]
Child parity	− 0.409***	− 0.136**	− 0.125*	− 0.113*
	[0.00]	[0.03]	[0.06]	[0.08]
*R*-squared adjusted	0.59	0.56	0.42	0.38
Dependent variable: health index				
Distance	− 0.067***	− 0.062***	− 0.053**	− 0.022*
	[0.00]	[0.00]	[0.03]	[0.08]
Mother’s age	− 0.910***	− 0.677***	− 0.846**	− 0.405***
	[0.01]	[0.01]	[0.02]	[0.01]
Mother’s education	0.967***	0.836***	0.955***	0.841***
	[0.01]	[0.01]	[0.00]	[0.01]
Mother’s ethnicity/race	0.078	0.073	0.077	0.069
	[0.22]	[0.25]	[0.22]	[0.29]
Child parity	0.509***	0.337***	0.148**	0.046
	[0.00]	[0.01]	[0.05]	[0.18]
*R*-squared adjusted	0.73	0.68	0.61	0.57
Comparison results: driver = distance				
	Distance			
	0–1 km	1–5 km	5–10 km	10–20 km
Total birth weight	− 0.084***	− 0.078*	− 0.048	− 0.025
	[0.00]	[0.07]	[0.14]	[0.29]
Low birth weight	0.056***	0.046***	0.039*	0.014
	[0.00]	[0.01]	[0.10]	[0.18]
Health index	− 0.067***	− 0.062***	− 0.053**	− 0.022*
	[0.00]	[0.00]	[0.03]	[0.08]

Figures in brackets denote *p* values. Standard errors are clustered on ZIP levels

***p ≤ 0.01

**p ≤ 0.05

*p ≤ 0.10

The results document that the effect of fracking wells on infants’ health at birth is negative in the cases where the health indicator is expressed through the infant’s total birth weight and the health index, while it turns out to be positive in the case where the health indicator is shown through the definition of low birth weight. Moreover, in relevance to the total birth weight, the results turn out to be significant only in the cases up to 5 km, with the stronger impact coming in the case where the distance is within 1 km. In relevance to the definition of total health index, the findings are significant across all distances, with the strongest impact occurring within the 1-km distance. For example, the coefficient − 0.067 within 1 km clearly indicates that births near a well site have a − 0.067 standard deviation decline in the health index. In relevance to the low birth weight, the findings highlight that the effect remains significant up to the 10-km distance, with the strongest value coming from the 1-km distance.

The lowest part of Table 1 reports some comparison results with respect to the primary control driver, which is the distance from the fracking well. The findings seem to provide consistent documentation to the adverse effect of fracking activities on infant mortality through the 5- to 10-km distance. However, it is only the overall health index for infants that explicitly illustrates that this adverse effect is statistically significant even in higher distances (10 to 20 km), indicating that fracking activities tend to have a more overall impact on infants’ health and not just on their weights. This raises important concerns about the implications for infants’ mortality rate in terms of how public health authorities respond to such adverse impacts. In particular, although records have explicitly shown that low birth weight figures clearly display a substantial risk factor for many negative outcomes in individuals’ lifetime, such as deficit hyperactivity disorders, asthma, lower test scores, lower schooling attainment, lower earnings, and higher rates of social welfare program participation (Black et al. 2007; Oreopoulos et al. 2008; Currie et al. 2017), others indicate that such hazardous activities can affect water and air quality, with further effects on mothers and subsequent impacts on infants (Vidic et al. 2013; Olmstead et al. 2013). As a result, there is an urgent need for tighter regulation not just from public health authorities, but also from environmental stakeholders so as to mitigate any potential threats to water and air quality. Finally, mothers should also receive support from mineral rights, which can confer a health benefit, partially offsetting any potential negative effects of fracking-related activities.

Table 2 repeats the analysis reported in Table 1, but this time, the examination period runs from 1996 to 2005. This analysis seems imperative so as to explore whether oil and gas drilling activities (based on regular/standard extracting methods) had any statistical impact on infants’ mortality rates. The regression considers the number of active wells that do not adopt any fracking technology, which was the majority of the cases (92.7%) prior to 2006. All other variables included remain the same. The new findings clearly indicate that drilling well activity has a very small impact on infants’ mortality which is statistically significant only at 10%, only with respect to the overall health index and in distances up to 5 km.

**Table 2:** Estimates of fracturing on infants’ health (1996–2005)

Variables	0–1 km	1–5 km	5–10 km	10–20 km
Dependent variable: total birth weight				
Distance	− 0.021	− 0.016	− 0.011	− 0.008
	[0.11]	[0.13]	[0.17]	[0.19]
Mother’s age	− 0.214**	− 0.227**	− 0.251**	− 0.244**
	[0.04]	[0.04]	[0.03]	[0.03]
Mother’s education	0.320***	0.334***	0.329***	0.358***
	[0.00]	[0.00]	[0.00]	[0.00]
Mother’s ethnicity/race	0.049	0.056	0.053	0.062
	[0.31]	[0.30]	[0.29]	[0.25]
Child parity	0.315***	0.369***	0.091**	0.259**
	[0.00]	[0.00]	[0.02]	[0.05]
*R*-squared adjusted	0.44	0.41	0.39	0.32
Dependent variable: low birth weight				
Distance	0.020	0.015	0.008	0.000
	[0.16]	[0.21]	[0.26]	[0.46]
Mother’s age	0.186**	0.195**	0.199**	0.205**
	[0.04]	[0.05]	[0.04]	[0.03]
Mother’s education	− 0.852***	− 0.859***	− 0.883***	− 0.828***
	[0.00]	[0.00]	[0.00]	[0.00]
Mother’s ethnicity/race	0.041	0.046	0.039	0.040
	[0.32]	[0.30]	[0.37]	[0.32]
Child parity	− 0.374***	− 0.188**	− 0.151**	− 0.132*
	[0.00]	[0.02]	[0.05]	[0.06]
*R*-squared adjusted	0.50	0.49	0.38	0.34
Dependent variable: health index				
Distance	− 0.053***	− 0.058***	− 0.028	− 0.019
	[0.01]	[0.01]	[0.12]	[0.16]
Mother’s age	− 0.877***	− 0.714***	− 0.768***	− 0.586***
	[0.00]	[0.00]	[0.00]	[0.00]
Mother’s education	0.884***	0.867***	0.852***	0.825***
	[0.00]	[0.00]	[0.00]	[0.00]
Mother’s ethnicity/race	0.064	0.069	0.071	0.062
	[0.27]	[0.23]	[0.22]	[0.28]
Child parity	0.492***	0.416***	0.377***	0.162*
	[0.00]	[0.00]	[0.01]	[0.07]
*R*-squared adjusted	0.68	0.63	0.62	0.55

Figures in brackets denote *p* values. Standard errors are clustered on ZIP levels

***p ≤ 0.01

**p ≤ 0.05

*p ≤ 0.10

Finally, after a referee’s recommendation, Table 3 repeats the empirical analysis, but this time, drilling activities are only associated with conventional (not fracking) methods. It is worth mentioning here that, on average, the conventional drilling activities accounted for the 28.9% over the period 2006–2017. The results document the very minor impact of such activities on infants’ birth rates and only with respect to the overall health index and in relevance to the 1-km distance from the (conventional) well. These findings provide supportive evidence to the substantial (negative) role of fracking drilling activities for infants’ health status.

**Table 3:** Estimates of conventional drilling on infants’ health (2006–2017)

Variables	0–1 km	1–5 km	5–10 km	10–20 km
Dependent variable: total birth weight				
Distance	− 0.012	− 0.005	− 0.001	− 0.000
	[0.20]	[0.34]	[0.46]	[0.68]
Mother’s age	− 0.196**	− 0.201**	− 0.224**	− 0.230**
	[0.05]	[0.05]	[0.04]	[0.03]
Mother’s education	0.286***	0.309***	0.311***	0.307***
	[0.00]	[0.00]	[0.00]	[0.00]
Mother’s ethnicity/race	0.038	0.044	0.049	0.053
	[0.40]	[0.32]	[0.28]	[0.24]
Child parity	0.302***	0.319***	0.166***	0.225***
	[0.00]	[0.00]	[0.01]	[0.00]
*R*-squared adjusted	0.32	0.28	0.23	0.18
Dependent variable: low birth weight				
Distance	0.011	0.006	0.000	0.000
	[0.29]	[0.40]	[0.57]	[0.72]
Mother’s age	0.162**	0.174**	0.183**	0.179**
	[0.05]	[0.05]	[0.04]	[0.04]
Mother’s education	− 0.629***	− 0.691***	− 0.654***	− 0.680***
	[0.00]	[0.00]	[0.00]	[0.00]
Mother’s ethnicity/race	0.036	0.041	0.033	0.038
	[0.39]	[0.34]	[0.42]	[0.37]
Child parity	− 0.329***	− 0.286***	− 0.198**	− 0.126*
	[0.00]	[0.01]	[0.03]	[0.06]
*R*-squared adjusted	0.41	0.37	0.39	0.31
Dependent variable: health index				
Distance	− 0.032**	− 0.017	− 0.013	− 0.008
	[0.05]	[0.12]	[0.20]	[0.29]
Mother’s age	− 0.658***	− 0.642***	− 0.589***	− 0.542***
	[0.00]	[0.00]	[0.00]	[0.00]
Mother’s education	0.705***	0.674***	0.627***	0.591***
	[0.00]	[0.00]	[0.00]	[0.00]
Mother’s ethnicity/race	0.047	0.034	0.023	0.014
	[0.39]	[0.50]	[0.63]	[0.81]
Child parity	0.427***	0.368***	0.311***	0.204**
	[0.00]	[0.00]	[0.00]	[0.04]
*R*-squared adjusted	0.50	0.44	0.39	0.32

Figures in brackets denote *p* values. Standard errors are clustered on ZIP levels

***p ≤ 0.01

**p ≤ 0.05

*p ≤ 0.10

## Causality tests

Table 4 and 5 shows the results of the Dumitrescu-Hurlin test. The results in panel A (with respect to the period 2006–2017) highlight that fracking wells Granger cause infants’ health at birth, while there is no reverse causality. In other words, there exist unidirectional causal relationship between fracking and infant’s health, with the direction of this relationship being only unilateral and only from the fracking side. Table 3 also reports the results (panel B) prior to the 2006 period, indicating the absence of any causality running from drilling activity to infants’ mortality and only in the case of the total health index and this at the 10% significance level.

**Table 4:** Dumitrescu-Hurlin Granger causality results

Tested hypothesis	Z-bar test
Panel A: 2006–2017	
Total birth weight	
H_0_: Fracking does not cause infants’ health at birth	9.864*** [0.00]
H_0_: Infants’ health at birth does not cause fracking	0.751 [0.24]
Low birth weight	
H_0_: Fracking does not cause infants’ health at birth	10.639*** [0.00]
H_0_: Infants’ health at birth does not cause fracking	0.682 [0.29]
Health index	
H_0_: Fracking does not cause infants’ health at birth	8.843*** [0.00]
H_0_: Infants’ health at birth does not cause fracking	0.661 [0.32]
Panel B: 1996–2005	
Total birth weight	
H_0_: Fracking does not cause infants’ health at birth	1.074 [0.18]
H_0_: Infants’ health at birth does not cause fracking	0.496 [0.49]
Low birth weight	
H_0_: Fracking does not cause infants’ health at birth	0.981 [0.22]
H_0_: Infants’ health at birth does not cause fracking	0.453 [0.60]
Health index	
H_0_: Fracking does not cause infants’ health at birth	1.372* [0.10]
H_0_: Infants’ health at birth does not cause fracking	0.482 [0.56]

Figures in brackets denote *p* values

***p ≤ 0.01

*p ≤ 0.10

**Table 5:** Panel unit root tests

H_0_: contains a unit root (CIPS test)	
Variables	Levels
Maternal births	
Total birth weight	− 4.996***
Low birth weight	− 5.109***
Health index	− 5.652***
Distance	− 5.728***
Mother’s age	− 6.637***

***p ≤ 0.01

## Conclusion

This study explored the impact of the fracking wells on infants’ birth health across Oklahoma counties. The findings indicated that the closer the mother’s residence at birth to fracking wells, the more negative are the effects on the infants’ birth health. The findings also documented the minor role of regular drilling activities in infants’ mortality prior to 2006, the threshold year that signified the importance of fracking activities in the case of the Oklahoma state. The results could be also explained through the impact of the fracking activities on the drinking quality index. These findings can motivate the rise of efficient regulatory actions with respect to both drinking water quality and to the shale gas industry.

State as well as federal policymakers need to seriously and considerable evaluate such significant public health costs associated with fracking activities. In other words, priority should be the prevention of public health costs, before considering any potential benefits associated with other dimensions of the economy. Overall, regulatory mechanisms are not that strong to effectively monitor potential negative effects on the population. Therefore, policymakers must cope with the potential risk to underscore the production of unconventional natural shale gas and oil, which does not seem to receive strong support today, or to develop it without considering any potential harms on the population’s health quality. A stronger regulatory environment is a high priority.
